# Maintenance treatment of combination with bevacizumab vs single agent for advanced non-squamous non-small cell lung cancer: A systematic review and meta-analysis

**DOI:** 10.1097/MD.0000000000026862

**Published:** 2021-08-06

**Authors:** Ying Kong, Liang Hong, Xiaocheng Xu, Jia Xu

**Affiliations:** Department of Oncology, The First People's Hospital of Xiaoshan, Hangzhou, China.

**Keywords:** bevacizumab, chemotherapy, maintenance therapy, monotherapy, non-squamous NSCLC, pemetrexed

## Abstract

**Background:**

When the patients of advanced non-squamous non-small cell lung cancer (NSCLC) have achieved remission by induction therapy, it is controversial that combination with bevacizumab is used as maintenance therapy. Pemetrexed is a classic drug for maintenance therapy, is bevacizumab the superiority to pemetrexed is also unclear. This meta-analysis aims to evaluate the effectiveness and safety of advanced non-squamous NSCLC in the maintenance treatment.

**Method:**

From the establishment as of December 6, 2020, PubMed, Embase, and Cochrane electronic databases were searched and the American Society of Clinical Oncology, European Society of Medical Oncology, and National Comprehensive Cancer Network databases in the past 10 years. The application of combination with bevacizumab, pemetrexed was studied in clinical trials of maintenance treatment for advanced NSCLC. The extracted data include progression-free survival (PFS), overall survival (OS), and grade 3–4 adverse events (AE).

**Results:**

Seven clinical trials we screened, 6 were phase III RCTs, and a cohort trial, including 3298 patients. Compared with bevacizumab and pemetrexed, PFS of combination with bevacizumab was significantly improved (hazard ratio [HR] = 0.71, 95% confidence interval [CI] = 0.65–0.77, *P* < .00001), but OS was not improved (HR = 0.93, 95% CI = 0.85–1.01, *P* = .10). Compared with bevacizumab and pemetrexed, no significant difference of PFS (HR = 0.87, 95% CI = 0.69–1.09, *P* = .21), and OS (HR = 0.87, 95% CI = 0.72–1.05, *P* = .15) was found. A higher incidence of grade 3–4 AE occurred in combination with bevacizumab (odds ratio = 1.63, 95% CI = 1.35–1.97, *P* < .00001).

**Conclusions:**

PFS was significantly improved in patients with advanced non-squamous NSCLC who use bevacizumab combination with single-agent as maintenance treatment, but it does not translate into the advantages of OS; compared with bevacizumab, no PFS and OS benefits were found. A higher incidence of grade 3–4 AE occurred in combination with bevacizumab than pemetrexed and bevacizumab.

## 1 Introduction

Lung cancer is a high incidence rate cancer and is also the most common cause of cancer death worldwide. Non-squamous histological types are the primary subtype of non-small cell lung cancer (NSCLC),^[1]^ accounting for 80% to 85% of lung cancer. At the time of advanced non-squamous NSCLC was diagnosed, systemic treatment including chemotherapy, immunotherapy, or targeted therapy can significantly prolong survival. If advanced non-squamous NSCLC of patients does not have driver gene mutations corresponding to existing specific inhibitors, platinum-based cytotoxic dual-drug chemotherapy is the basic plan for initial systemic treatment.^[2]^ Bevacizumab is an anti-vascular endothelial growth factor antibody, based on the combined regimen, bevacizumab was used, and the objective response rate, progression-free survival (PFS), and overall survival (OS) were better than chemotherapy alone.^[3,4]^ Objective remission was received after 4 to 6 cycles of initial therapy. Continued maintenance treatment can make the patient obtain a longer lifetime.

After initial treatment of advanced NSCLC, pemetrexed, docetaxel, gemcitabine, and bevacizumab can significantly prolong PFS as single-agent maintenance therapy. The JMEN trial compared pemetrexed with placebo showed that both PFS and OS were significantly improved.^[5]^ However, there are no randomized trials that directly compare these 3 drugs as maintenance therapy. Bevacizumab plus carboplatin and pemetrexed/paclitaxel are approved for the first-line treatment of metastatic non-squamous NSCLC.^[6]^ After treatment with pemetrexed and bevacizumab regimens, 1 of these drugs can continue to be used for maintenance therapy. The National Comprehensive Cancer Network Guidelines recommend using pemetrexed, bevacizumab, or pemetrexed plus bevacizumab as a maintenance treatment for patients with advanced NSCLC who have achieved remission by induction therapy. However, the combination of bevacizumab with a drug for maintenance therapy is controversial. AVAPERL trial compared bevacizumab combined with pemetrexed and bevacizumab alone as maintenance therapy. However, the OS of the bevacizumab plus pemetrexed group was extended by 4 months, the difference was not statistically significant.^[7]^ Point Break trial found that the difference was not statistically significant in OS, although the PFS of the bevacizumab plus pemetrexed group was longer.^[8]^

Therefore, the meta-analysis of 6 randomized clinical trials (RCTs) and 1 cohort study aims to study the efficacy and safety of the combination of bevacizumab vs bevacizumab or pemetrexed and pemetrexed vs bevacizumab in the maintenance treatment of non-squamous NSCLC.

## 2 Materials and methods

### 2.1 Search strategy

From the establishment as of December 6, 2020, PubMed, Embase, and Cochrane electronic databases were searched, National Comprehensive Cancer Network, American Society of Clinical Oncology (ASCO), and European Society of Medical Oncology from 2010 to 2020 Database, (CENTRAL) publishes relevant clinical trials. Strictly abide by the “Private Reporting Project for Systematic Reviews and Meta-Analysis” (PRISMA) Statement Guidelines 2009.^[9]^ The following keywords were applied: bevacizumab, pemetrexed, chemotherapy, monotherapy, NSCLC, and maintenance therapy.

### 2.2 Inclusion criteria

(1) Population: >18 years of age diagnosed as advanced non-squamous NSCLC by pathology; (2) intervention: 4 to 6 cycles of induction chemotherapy, bevacizumab combined with single drug (cytotoxic drug, EGFR-TKI, etc) or pemetrexed or bevacizumab monotherapy for maintenance treatment; (3) results: hazard ratio (HR) of PFS and OS, odds ratio (OR) of grade 3–4 adverse events (AE); and (4) study design: main screen RCTs.

### 2.3 Data extraction

The following data from each eligible study were extracted independently by 2 reviewers (KY and HL): the surname and year of publication of the first author, trial phase, the number of patients, the median age, induction, and maintenance therapy drugs, HR of PFS and OS, the number of occurrences of grade 3–4 AE. All the differences shall be resolved by consensus or through consultation with the third judge.

### 2.4 Assess the risk of bias and assess the quality of evidence

Following the guidelines in the Cochrane Handbook for bias risk assessment.^[10]^ Two researchers objectively reviewed all studies, and assigned values of the following 6 areas: random sequence generation, the assignment is hidden, participants, and personnel are blind, result evaluation is blind, result data is incomplete, selective reporting, and other biases. In the blindness of researchers and participants (performance bias) and the blindness of result evaluation (detection bias), all open trials were identified as “high risk.” Four levels to assess the quality of evidence by the GRADE system: high, moderate, low, and very low.^[11]^

### 2.5 Statistical analysis

Bevacizumab combined with pemetrexed or erlotinib vs bevacizumab or pemetrexed: bevacizumab combined with pemetrexed or erlotinib as the experimental group, bevacizumab or pemetrexed is the control group; pemetrexed vs bevacizumab: pemetrexed is the test group, and bevacizumab is the control group. We estimated the HR and 95% confidence interval (CI) of PFS and OS, and the OR and 95% CI of grade 3–4 AE in the 2 groups. A random-effects model is used if there is moderate heterogeneity; otherwise, choose to use the fixed effects model. A subgroup analysis or sensitivity analysis is performed if significant heterogeneity is identified. The Cochran *Q* test and *I*^2^ statistics were used to assess the heterogeneity between studies. To assess potential publication bias, a funnel plot and Egger weighted linear regression test was used. All statistical data analysis and the risk of bias graphics are performed using Review Manager 5.3. GRADE profiler software (version 3.6) is used to assess the level of evidence. All *P* values are bidirectional and are considered statistically significant at the .05 level.

### 2.6 Ethical approval

Since this study is on the basis of published articles and do not involve patients, ethical approval and informed consent of patients are not required.

## 3 Results

**Figure 1:**
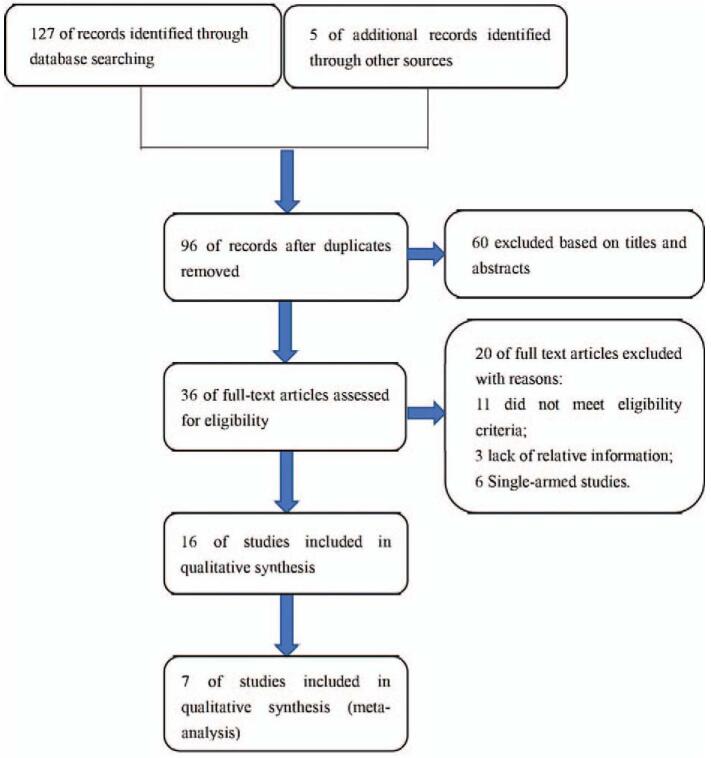
Flow chart of the literature selection.

Figure 1 shows the literature screening process. We initially searched PubMed, Embase, and Cochrane to identify 127 potential full-text articles. Five full-text articles were from ASCO, European Society of Medical Oncology, and National Comprehensive Cancer Network databases. One hundred twenty-five articles were excluded according to the inclusion criteria. Finally, 7 qualified articles included PFS, OS, and 3–4 grade AE data,^[7,8,12–16]^ 2 of which are from ASCO conference reports in the last 2 years.^[15,16]^

Tables 1 and 2 list the main characteristics of the 7 clinical trials. Six clinical trials are phase III RCTs,^[7,8,12,14–16]^ and 1 clinical trial is a cohort study.^[13]^ The pathological type of the patient was non-squamous NSCLC, the stage IIIB-IV, and the physical status score was 0 to 1; the total number of patients was 3299, of which 1441 were female, and 1858 were male. The median age of the patients is 63.2 years old (range 38–79). Among them, 4 trials were bevacizumab + pemetrexed vs bevacizumab,^[7,8,15–16]^ 1 trial was bevacizumab + pemetrexed vs pemetrexed,^[13]^ and 1 trial was bevacizumab + erlotinib vs bevacizumab,^[12]^ and the 2 trials are pemetrexed vs bevacizumab.^[14,15]^ PFS, OS, grade 3–4 AEs were reported in 7 trials, and the HR and 95% CI of PFS and OS were directly obtained; we conducted a subgroup analysis of grade 3–4 AEs and screened the main 7 items. The indicators were neutropenia, anemia, thrombocytopenia, hypertension, proteinuria, embolism, and hemorrhage. The corresponding OR and 95% CI were calculated based on the number of patients with grade 3–4 AE in the 2 groups in the trials.

**Table 1:** Characteristics of included 6 randomized controlled trials and 1 cohort study.

			Sample size		Median age (range)		Male/female	
First author	Year	Trial phase	Trial	control	Trial	control	Trial	Control
Bruce et al^[12]^	2013	IIIB	370	373	64 (31–88)	64 (23–83)	193/177	196/177
Fabrice et al^[7]^	2013	III	125	120	NM	NM	72/53	68/52
Jyoti et al^[8]^	2013	III	292	298	63.8	64.3	148/144	159/156
Domenico et al^[14]^	2015	III	58	60	62 (41–71)	60 (35–72)	42/18	45/13
Oliver et al^[13]^	2016	II	77	52	61.6 (32.3–76.5)	63.2 (38.2–79)	45/32	24/28
Ramalingam et al^[15]^	2019	III	293/294	287	64/63	65	143/140143/140	150/147151/147
Seto et al^[16]^	2020	III	298	301	65 (32–81)	65 (27–81)	221/78	209/86

**Table 2:** Maintenance treatment regimens of 7 studies, and HR with 95% CI for mPFS and mOS.

			Therapy		HR (95%CI)	
First author	Year	Design	Trial	Control	mPFS	mOS
Bruce et al^[12]^	2013	RCT	Bevacizumab + erlotinib	Bevacizumab	0.708 (0.58–0.864)*P* = .001	0.917 (0.698–1.205)*P* = .534
Fabrice et al^[7]^	2013	RCT	Bevacizumab + pemetrexed	Bevacizumab	0.48 (0.35–0.66)*P* = .001	0.75 (0.47–1.19)*P* = .219
Jyoti et al^[8]^	2013	RCT	Bevacizumab + pemetrexed	Bevacizumab	0.83 (0.71–0.96)*P* = .012	1.0 (0.86–1.16)*P* = .949
Domenico et al^[14]^	2015	RCT	Pemetrexed	Bevacizumab	0.79 (0.53–1.17)*P* = .24	0.93 (0.60–1.42)*P* = .73
Oliver et al^[13]^	2016	Cohort study	Bevacizumab + pemetrexed	Pemetrexed	0.7 (0.5–1.0)*P* < .041	1.0 (0.7–1.6)*P* = .890
Suresh et al^[15]^	2019	RCT	Bevacizumab + pemetrexed/pemetrexed	Bevacizumab	0.67 (0.55–0.82)*P* = .001;0.905 (0.69–1.03)*P* = .06	0.9 (0.73–1.12)*P* = .280.86 (0.70–1.07)*P* = .12
Takashi et al^[16]^	2020	RCT	Bevacizumab + pemetrexed	Bevacizumab	0.67 (0.57–0.79)*P* = .001	0.87 (0.73–1.05)*P* = .069

The 3 forest maps list the results of the risk of bias. Six RCTs were randomly sequenced^[7,8,12,14–16]^ and 2 studies were open random allocation.^[8,13]^ One study proved sufficient blinding^[12]^ and 5 studies did not have blinding. Still, the author of this article determined that the outcome is unlikely to be affected by the lack of blinding,^[8,13–16]^ and 1 study could not determine whether there was blinding.^[7]^ The study protocol is available for 7 trials, and all pre-declared outcomes have been reported. Six studies did not find other biases, and 1 study did not have enough information to evaluate whether there were significant biases.^[8]^ All included studies have a low to moderate risk of bias and are of sufficiently high quality according to the Jadad scoring tool (Fig. 5).

**Figure 2:**
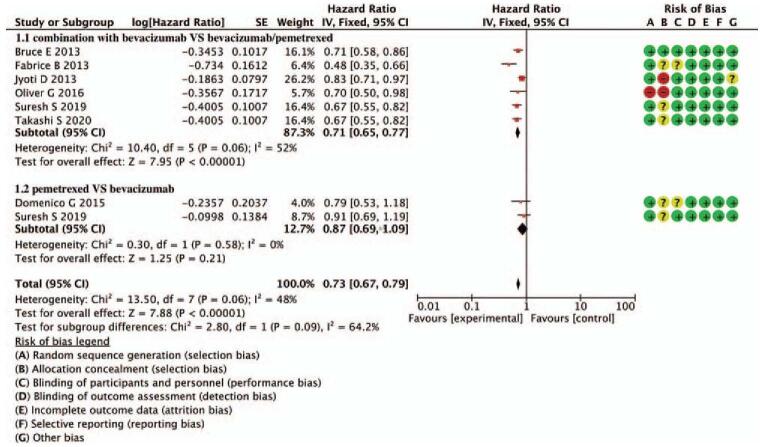
Forest plot of merged analyses for HR with 95%CI for mPFS. CI = confidence interval, HR = hazard ratio, mPFS = median PFS.

Figure 2 shows the PFS analysis. All 7 studies reported available data on PFS. The median PFS (mPFS) of combination with bevacizumab vs pemetrexed was 6.5, 4.1 months, respectively (HR = 0.71, 95% CI = 0.65–0.77, *P* < .00001), indicating combination with bevacizumab can significantly prolong PFS. The mPFS of pemetrexed vs bevacizumab was 6.6, 6.3 months, respectively (HR = 0.87, 95% CI = 0.69–1.09, *P* = .21), between the 2 groups, no significant difference was found. The combined HR of the 2 sub-combinations was 0.73, 95% CI = 0.67–0.79, *P* < .00001, the benefit of PFS was derived from the combination with the bevacizumab subgroup. There was moderate heterogeneity in the 2 groups (*P* = .06, *I*^2^ = 48%), and the inhibitory quality was derived from the different treatment options between the 2 groups.

**Figure 3:**
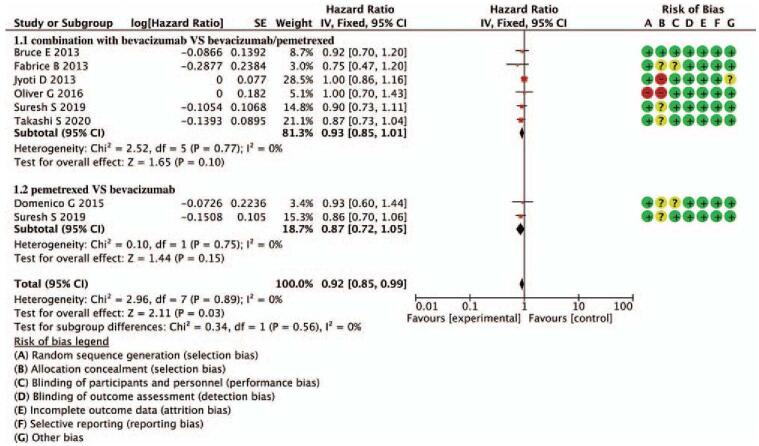
Forest plot of merged analyses for HR with 95%CI for mOS. CI = confidence interval, HR = hazard ratio, mOS = median OS.

Figure 3 shows the OS analysis. All 7 studies reported available data on OS. The mOS of combination with bevacizumab vs pemetrexed was 14.4, 13.9 months, respectively (HR = 0.93, 95% CI = 0.85–1.01, *P* = .10). The mOS of pemetrexed vs bevacizumab was 15 and 14.4 months, respectively (HR = 0.87, 95% CI = 0.72–1.05, *P* = .15), so the 2 subgroups have no significant difference. Neither pemetrexed vs bevacizumab nor combination with bevacizumab vs bevacizumab /pemetrexed were found an advantage in OS. Combined the 2 subgroups (HR = 0.92, 95% CI = 0.85–0.99, *P* = .03), and no heterogeneity was found in the 2 subgroups (*P* = .56, *I*^2^ = 0%).

**Figure 4:**
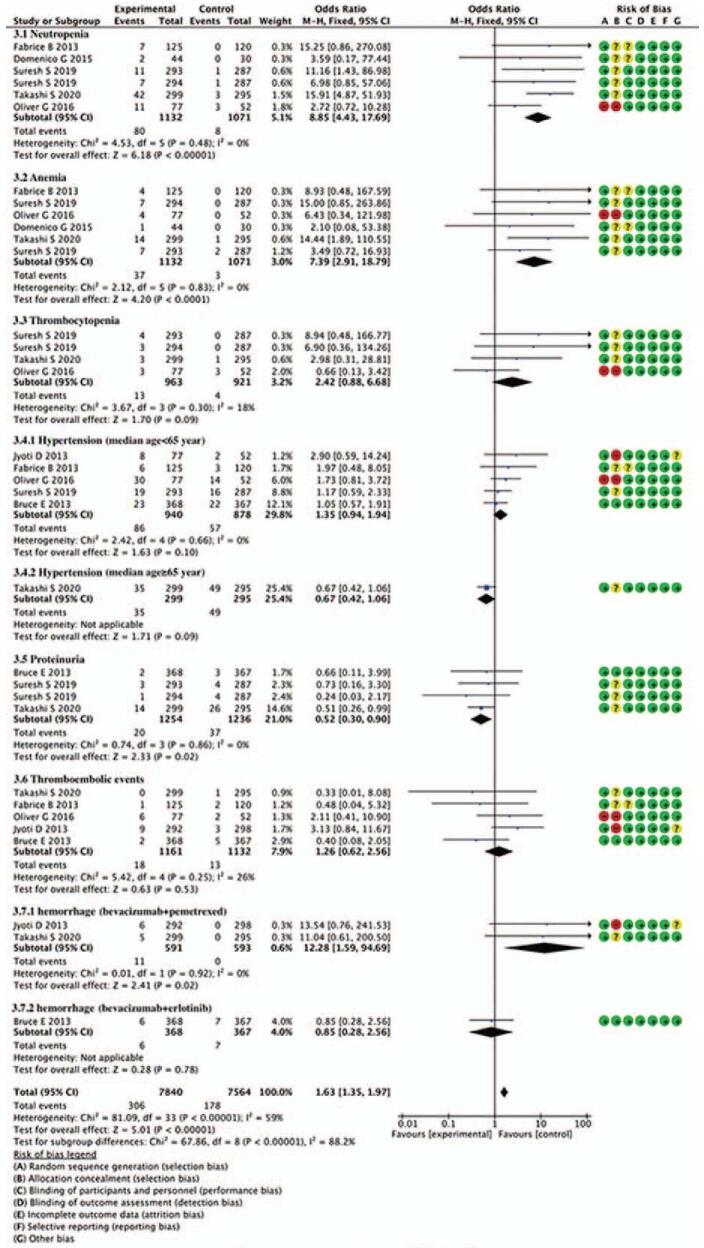
Forest plot of merged analyses for OR with 95%CI for the incidence of grade 3–4 AE. AE = adverse events, CI = confidence interval, OR = odds ratio

Figure 4 shows the analysis of grade 3–4 AE; the results show that combination with bevacizumab vs bevacizumab/pemetrexed, pemetrexed vs bevacizumab. The incidence of neutropenia and anemia was higher in combination with bevacizumab and pemetrexed. The combined OR was (8.85, 95% CI = 4.43–17.69, *P* < .00001), (7.39, 95%CI = 2.91–18.79, *P* < .0001), respectively. The incidence of thrombocytopenia was not significantly different (OR = 2.42, 95% CI = 0.88–6.68, *P* = .09). Combination with bevacizumab vs bevacizumab/pemetrexed. The incidence of hypertension (OR = 1.35, 95% CI = 0.94–1.94, *P* = .1) and thromboembolic events (OR = 1.26, 95% CI = 0.62–2.56, *P* = .53) was not significantly different; the incidence of proteinuria in bevacizumab was higher (OR = 0.59, 95% CI = 0.35–0.98, *P* = .04); the incidence of hemorrhage in the combination with bevacizumab group was higher (OR = 12 .28, 95% CI = 1.59–94.69, *P* = .02).

A sensitivity analysis was performed by deleting individual trials to assess the stability of the results, and no separate study changed the combined results of PFS and OS. Combination with bevacizumab can significantly improve PFS, but OS between the 2 groups is not significantly different. Compared with pemetrexed and bevacizumab, no PFS and OS advantages were found. For this meta-analysis, the results of PFS and OS are stable. The PFS and OS of all 7 studies were displayed in a funnel chart to evaluate the reliability of our results. The funnel chart shows symmetry, and no evidence of publication bias was observed (*P* > .05) (Fig. 6).

**Figure 5:**
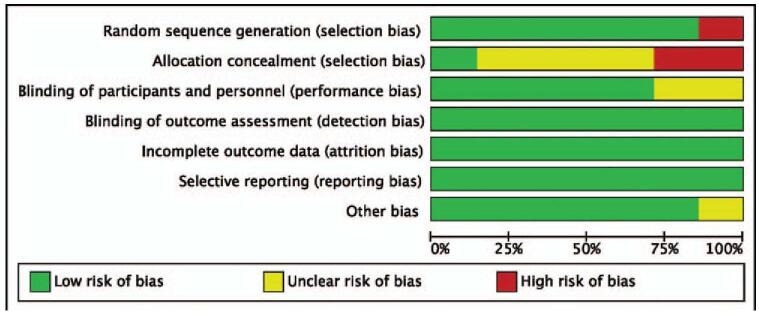
Assessment of the quality of the included studies: low risk of bias (green hexagons), unclear risk of bias (yellow hexagons), and high risk of bias (red hexagons).

**Figure 6:**
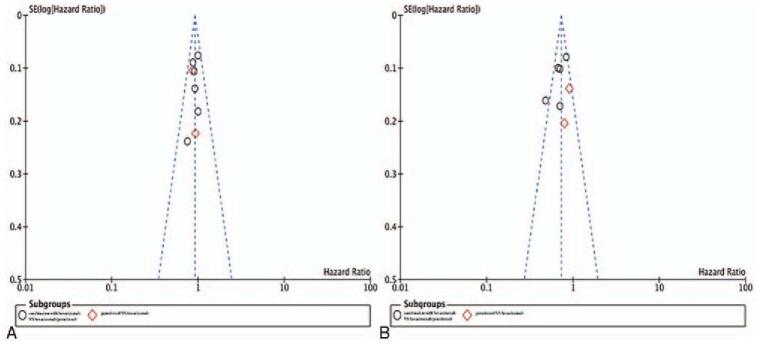
Publication bias analysis by funnel plot graphic. (a) PFS. (b) OS. OS = overall survival, PFS = progression-free survival

## 4 Discussion

In this meta-analysis, we analyzed the efficacy and safety of combination with bevacizumab, pemetrexed, and bevacizumab in the maintenance treatment of advanced non-squamous NCSLC. There are no restrictions on the expression of EGFR, PD-L1, etc. Our data show that combination with bevacizumab (pemetrexed, erlotinib) can significantly improve PFS, but it does not translate into an OS advantage. In contrast, pemetrexed is not significantly more effective than bevacizumab for PFS and OS.

ECOG4599 and AVAIL studies have shown that bevacizumab combined with chemotherapy and continued bevacizumab maintenance therapy significantly prolonged the patient's PFS.^[17,18]^ In addition to anti-angiogenic drugs, maintenance therapy with chemotherapeutic drugs can also improve the prognosis. Single-agent maintenance of docetaxel and gemcitabine can also prolong PFS,^[19]^ but adverse reactions limit its use. Pemetrexed is a highly effective and tolerable good advantage, and previous studies of PARAMOUNT have also confirmed that pemetrexed as maintenance therapy can improve PFS in advanced NCSLC. However, the combination of the 2 drugs as maintenance therapy is controversial, and it is unclear whether pemetrexed alone is better than bevacizumab alone as maintenance therapy. The AVPEARL study showed that combination with bevacizumab could significantly improve PFS. Although OS is superior to bevacizumab alone in the trend,^[7]^ it is statistically insignificant and may be related to clinical design. The number of included cases is not enough to find the difference between the 2 groups. ECOG5508 study showed that the combination of pemetrexed and bevacizumab was not superior to pemetrexed alone or bevacizumab alone.^[15]^ There has not been a meta-analysis to compare 2-drug combinations, including bevacizumab and pemetrexed versus bevacizumab in non-squamous NCSLC about maintenance treatment. The results of our meta-analysis may provide some reference for the maintenance treatment of advanced non-squamous NCSLC.

Among the 7 clinical trials we screened, Swiss Group for Clinical Cancer Research is a non-randomized phase II clinical study with 2 stratifications.^[13]^ Although the treatment allocation was not randomized, the baseline characteristics were balanced, and the remaining 6 clinical trials all are RCTs. The 7 clinical trial induction programs were bevacizumab + pemetrexed/paclitaxel + cisplatin/carboplatin. It is recommended to use 4 to 6 cycles of platinum-based initial therapy for patients with advanced non-squamous cell NSCLC in good physical condition. Prolonging the cycle will increase toxicity and only slightly improve survival.^[20,21]^ Gruppo Oncologico Italia Meridionale is 6 cycles of chemotherapy of the induction therapy in 7 clinical trials, the rest are 4 cycles.

Seven clinical trials were divided into 2 subgroups for PFS and OS analysis according to different maintenance treatment plans. The first subgroup is bevacizumab combined with pemetrexed/erlotinib vs bevacizumab/pemetrexed, which was found to be moderately heterogeneous in PFS analysis (*I*^2^ = 52%); the heterogeneity comes from AVAPERL, the dose of bevacizumab in this trial is 7.5 mg/kg. In comparison, the dose of bevacizumab in the other 4 clinical studies is 15 mg/kg, after removing this clinical trial, the heterogeneity dropped to 5%, but did not change the overall result (*P* < .0001). No heterogeneity was found in pemetrexed vs bevacizumab; no statistically significant difference was found between the 2 subgroups, but the weight of the 2 clinical trials is small, so the benefit of PFS comes from bevacizumab combined with pemetrexed/erlotinib vs bevacizumab/pemetrexed group. No heterogeneity was found in OS in the 2 subgroups (*I*^2^ = 0%).

For patients with advanced NSCLC at the time of presentation, it should be evaluated whether there are somatic driver gene mutations, such as EGFR, ALK, ROS1, and BRAFV600E mutations, and whether express programmed cell death ligand 1 (PD-L1). This information should be used to guide the selection of initial treatment (chemotherapy vs molecularly targeted drugs vs immunotherapy). This information can also help guide maintenance treatment. Erlotinib is an EGFR tyrosine kinase inhibitor, PFS and OS can be improved as maintenance therapy both in patients with EGFR activating mutations and unselected patients,^[22,23]^ but wild-type EGFR patients of the evidence of PFS benefit is inconsistent. The ATLAS trial evaluated the effectiveness and safety of bevacizumab combined with erlotinib versus bevacizumab after 4 cycles of induction therapy with bevacizumab combined with chemotherapy.^[12]^ The enrolled patients did not know the EGFR status. After EGFR biomarker analysis, the results showed that patients with EGFR mutations in bevacizumab combined with erlotinib benefited from PFS, but OS did not improve. Besides, 3 clinical trials ruled out EGFR mutation, and 3 clinical trials did not clarify EGFR status. There may be a small part of EGFR mutations patients. COMPASS study showed that the OS of the bevacizumab + pemetrexed group was extended by 3.5 months,^[16]^ but no statistically significant difference was found. It was found that bevacizumab + pemetrexed could prolong the OS and PFS in the subgroup analysis. The effect of EGFR inhibitors on those without EGFR activating mutations is unknown, whether it is used as first-line treatment, maintenance therapy, or second-line treatment. The expression of PD-L1 in 7 clinical trials is unknown, and there may be some positive PD-L1 expression patients. For negative driver genes and unknown PD-L1 expression, pembrolizumab combined with pemetrexed and carboplatin for the first-line treatment of advanced non-squamous NCSLC has been approved by the US FDA. In on-squamous NSCLC patients, regardless of the PD-L1 expression, compared with bevacizumab + chemotherapy, the checkpoint inhibitor atezolizumab combined with bevacizumab/chemotherapy is more effective.^[24]^ Still, due to related side effects, it is not a preferred solution. There is currently no study comparing platinum combined with bevacizumab and combined with pembrolizumab head-to-head. In the maintenance treatment stage, pembrolizumab is usually continued until the disease progresses. There is no direct comparison of whether pembrolizumab is more advantageous than bevacizumab or bevacizumab combination therapy.

In our meta-analysis, grade 3–4 neutropenia, anemia, and hemorrhage have a higher incidence in the combination therapy of bevacizumab and pemetrexed; no significant differences of thrombocytopenia, thromboembolic events, and hypertension were found. Five of the 7 clinical trials did not exclude brain metastases. The data on patients with brain metastases treated with adequate anticoagulation showed that the use of bevacizumab is safe. There is no evidence that bevacizumab increases the risk of a cerebral hemorrhage.^[25,26]^ However, the risk of severe toxicity may increase in older adults. Hypertension is a common complication of bevacizumab, and meta-analysis shows that bevacizumab combination therapy does not increase the risk of grade 3–4 hypertension. Compared with combination with bevacizumab and pemetrexed, the incidence of grade 3–4 proteinuria increased slightly, and the overall incidence of mild proteinuria in patients treated with bevacizumab was 21% to 63%, but about 2% of treated patients have grade 3–4 proteinuria. Compared with single-agent therapy, combination with bevacizumab will generally increase grade 3–4 adverse reactions. More clinical trials comparing pemetrexed and bevacizumab are needed to verify their grade 3–4 adverse reactions reaction.

This meta-analysis has certain limitations. One is a non-randomized phase II clinical trial, which has a random allocation sequence and allocation concealed bias. In addition, the patients who received maintenance treatment in the Point Break study were randomized and induced. There is a limit to the possibility of induction therapy affecting the maintenance treatment plan. Only 1 clinical trial has clarified blinding, and there may be bias in blinding. Among the 7 clinical trials, 3 clinical trials did not describe the EGFR status, and the PD-L1 expression in the 7 clinical trials was unknown. Some patients may have EGFR mutations or positive PD-L1 expression, which may affect the research results. These trials have different treatment options, so the grouping meta-analysis only included a limited number of studies. Gruppo Oncologico Italia Meridionale does not use PFS and OS as the primary endpoints, so the sample size is small. In hematological toxicity analysis, the clinical sample size is small, and more clinical data are needed.

In conclusion, our meta-analysis showed that combination with bevacizumab could significantly improve PFS in the maintenance treatment of non-squamous NSCLC, but it does not translate into the OS’ advantage; pemetrexed and bevacizumab compared with bevacizumab, no benefits of PFS and OS were found. The combination with the bevacizumab group and the pemetrexed group have a higher incidence of neutropenia, anemia, and hemorrhage (grade 3–4), and the bevacizumab group has a higher incidence of proteinuria (grade 3–4). In the incidence of thrombocytopenia, hypertension, and thromboembolic events (grade 3–4), no significant difference was found. Therefore, combination with bevacizumab is not recommended due to the lack of OS benefit and higher adverse reactions; bevacizumab is not more advantageous than pemetrexed. Due to the lack of the literature, further verification is needed.

## Author contributions

**Conceptualization:** Ying Kong, Liang Hong.

**Data curation:** Ying Kong, Liang Hong, Jia Xu.

**Formal analysis:** Ying Kong, Liang Hong.

**Investigation:** Ying Kong, Liang Hong, Jia xu.

**Methodology:** Ying Kong, Liang Hong, Xiaocheng Xu.

**Writing – original draft:** Ying Kong.

**Writing – review & editing:** Ying Kong, Liang Hong, Xiaocheng Xu.
